# Humoral and Cellular Immunogenicity of Six Different Vaccines against SARS-CoV-2 in Adults: A Comparative Study in Tunisia (North Africa)

**DOI:** 10.3390/vaccines10081189

**Published:** 2022-07-27

**Authors:** Melika Ben Ahmed, Hedia Bellali, Mariem Gdoura, Imen Zamali, Ouafa Kallala, Ahlem Ben Hmid, Walid Hamdi, Hela Ayari, Hajer Fares, Karim Mechri, Soumaya Marzouki, Henda Triki, Nissaf Ben Alaya, Mohamed Kouni Chahed, Anis Klouz, Sonia Sebai Ben Amor, Chiheb Ben Rayana, Myriam Razgallah Khrouf, Chokri Hamouda, Noomene Elkadri, Riadh Daghfous, Abdelhalim Trabelsi

**Affiliations:** 1 Laboratory of Clinical Immunology, Pasteur Institute of Tunis, Tunis 1002, Tunisia; melika.benahmed@pasteur.tn (M.B.A.); imen.zamali@fmt.utm.tn (I.Z.); ahlem.benhmid@fmt.utm.tn (A.B.H.); walid.hamdi@pasteur.tn (W.H.); karimmechri91@gmail.com (K.M.); soumaya.marzouki@pasteur.tn (S.M.); 2 Faculty of Medicine of Tunis, Tunis El Manar University, Tunis 1068, Tunisia; hedia.bellali@fmt.utm.tn (H.B.); henda.triki@pasteur.tn (H.T.); nissaf.bouafif@rns.tn (N.B.A.); mohamed.chahed@fmt.rnu.tn (M.K.C.); anis.klouz@rns.tn (A.K.); chokri.hamouda@fmt.utm.tn (C.H.); noomene.elkadri@rns.tn (N.E.); riadh.daghfous@rns.tn (R.D.); 3 Department of Clinical Epidemiology, Habib Thameur Hospital, Tunis 1008, Tunisia; 4 Laboratory of Virology, Pasteur Institute of Tunis, Tunis 1002, Tunisia; mariem.gdoura@pasteur.tn; 5 Faculty of Pharmacy, University of Monastir, Monastir 5000, Tunisia; ouafa.kallala@rns.tn (O.K.); ayari_hela@yahoo.fr (H.A.); hajerfares@gmail.com (H.F.); chiheb.rayana@rns.tn (C.B.R.); dpm.dg@rns.tn (M.R.K.); 6 Laboratory of Virology, Sahloul University Hospital, Sousse 4002, Tunisia; 7 National Observatory of New and Emerging Diseases, Tunis 1002, Tunisia; 8 National Drug Control Laboratory, Tunis 1082, Tunisia; sonia.sebaibenamor@rns.tn; 9 Department of Pharmacy, National Centre of Bone Marrow Transplantation, Tunis 1006, Tunisia; 10 National Instance for Evaluation and Accreditation in Health, Tunis 1002, Tunisia; 11 Medical Research Directorate, Ministry of Health of Tunisia, Tunis 1006, Tunisia; 12 National Pharmacovigilance Center, Tunis 1006, Tunisia

**Keywords:** COVID-19, vaccines, humoral immunity, cellular immunity

## Abstract

Background: The mass vaccination campaign against SARS-CoV-2 was started in Tunisia on 13 March 2021 by using progressively seven different vaccines approved for emergency use. Herein, we aimed to evaluate the humoral and cellular immunity in subjects aged 40 years and over who received one of the following two-dose regimen vaccines against SARS-CoV-2, namely mRNA-1273 or Spikevax (Moderna), BNT162B2 or Comirnaty (Pfizer-BioNTech), Gam-COVID-Vac or Sputnik V (Gamaleya Research Institute), ChAdOx1-S or Vaxzevria (AstraZeneca), BIBP (Sinopharm), and Coronavac (Sinovac). Material and methods: For each type of vaccine, a sample of subjects aged 40 and over was randomly selected from the national platform for monitoring COVID-19 vaccination and contacted to participate to this study. All consenting participants were sampled for peripheral blood at 3–7 weeks after the second vaccine dose to perform anti-S and anti-N serology by the Elecsys^®^ (Lenexa, KS, USA) anti-SARS-CoV-2 assays (Roche^®^ Basel, Switzerland). The CD4 and CD8 T cell responses were evaluated by the QuantiFERON^®^ SARS-CoV-2 (Qiagen^®^ Basel, Switzerland) for a randomly selected sub-group. Results: A total of 501 people consented to the study and, of them, 133 were included for the cellular response investigations. Both humoral and cellular immune responses against SARS-CoV-2 antigens differed significantly between all tested groups. RNA vaccines induced the highest levels of humoral and cellular anti-S responses followed by adenovirus vaccines and then by inactivated vaccines. Vaccines from the same platform induced similar levels of specific anti-S immune responses except in the case of the Sputnik V and the AstraZeneca vaccine, which exhibited contrasting effects on humoral and cellular responses. When analyses were performed in subjects with negative anti-N antibodies, results were similar to those obtained within the total cohort, except for the Moderna vaccine, which gave a better cellular immune response than the Pfizer vaccine and RNA vaccines, which induced similar cellular immune responses to those of adenovirus vaccines. Conclusion: Collectively, our data confirmed the superiority of the RNA-based COVID-19 vaccines, in particular that of Moderna, for both humoral and cellular immunogenicity. Our results comparing between different vaccine platforms in a similar population are of great importance since they may help decision makers to adopt the best strategy for further national vaccination programs.

## 1. Introduction

The SARS-CoV-2 virus pandemic, triggered in China in December 2019, quickly reached the five continents [1]. Tunisia rapidly put in place a plan to fight this threat [2]. The first case was notified on 3 March 2020 and since then the epidemic has continued to spread throughout the country [3]. Around the world, several pharmaceutical companies have started research with the aim of manufacturing a vaccine that would help to slow the momentum of the epidemic.

On 2 October 2020, a technical advisory committee was created within the Ministry of Health in Tunisia whose main mission was to monitor the progress in research and development of new vaccines against SARS-CoV-2 in order to provide decision-makers with the best vaccine platforms for subsequent acquisition. Thus, and in a progressive manner, several vaccines were approved for emergency use and the mass vaccination campaign was started on 13 March 2021. One of the great features of this campaign was the opportunity to use seven different COVID-19 vaccines.

As of 12 January 2022, 12,445,869 doses have been distributed in Tunisia with 6,069,911 subjects having received a complete vaccination with one of the seven vaccines been used, namely, two doses of mRNA-1273 or Spikevax (Moderna, Cambridge, MA, USA), BNT162B2 or Comirnaty (Pfizer-BioNTech, New York, NY, USA), Gam-COVID-Vac or Sputnik V (Gamaleya Research Institute, Moscow, Russia), ChAdOx1-S or Vaxzevria (Astrazeneca, Cambridge, UK), BIBP (Sinopharm, Beijing, China) and Coronavac (Sinovac, Beijing, China) or one dose of Janssen vaccine (Ad26COV2.S).

Given the development of knowledge on the real-life efficacy of the various vaccines marketed around the world [4], the results of fragmentary studies on the immunogenicity of these vaccines, which showed quite significant differences, and the increasingly frequent observation of infections on vaccinated sites, it turned out to be essential to evaluate on scientific bases and on consequent samples the humoral and cellular immunogenicity of vaccines used in the country. This study would help the steering committee to adopt the best strategy for better vaccination coverage and better protection of the population. Indeed, this cross-sectional study would allow us to refine the number of doses really essential for each vaccine, to offer the possibility of interchangeability between the platforms and to better adapt the used vaccine, if necessary, according to the profiles of the subjects and the vaccine availability.

Herein, we aimed to evaluate the humoral and cellular immunity in subjects aged 40 years and over and vaccinated in Tunisia by one of the six vaccines against SARS-CoV-2 used in a two-dose regimen.

## 2. Materials and Methods

### 2.1. Ethic Statement

The study was approved by the PPC (Personal Protection Committee) ethics committee of the Center, Tunisia (number TN2021-NAT-INS-71). All patients provided written informed consent for the collection of samples and subsequent analysis. All research was conducted according to the declaration of Helsinki principles.

### 2.2. Study Population

This was a cross-sectional study on a sample of the Tunisian population chosen at random from vaccinated subjects aged 40 and over. The following inclusion and exclusion criteria were used:

Inclusion Criteria
  - Subjects aged 40 and over
  - Having received two doses of one of the following vaccines mRNA-1273 or Spikevax (Moderna), BNT162B2 or Comirnaty (Pfizer-BioNTech), Gam-COVID-Vac or Sputnik V (Gamaleya Research Institute), ChAdOx1-S or Vaxzevria (Astrazeneca), BIBP (Sinopharm) and Coronavac (Sinovac)
  - Vaccinated at one of the vaccination centers of the governorates of Tunis, Nabeul, Bizerte, Sousse, Monastir, and Mahdia
  - Having given informed consent

Exclusion Criteria
  - Subjects having had symptomatic COVID-19 before or after vaccination.
  - Pregnant women
  - Immunocompromised patients or under immunosuppressive treatments.

In total, 2789 subjects aged 40 and over who met the inclusion criteria were chosen randomly from the six governorates through the evax.tn App database (https://evax.tn accessed on 29 July 2021), the national Tunisian platform for monitoring COVID-19 vaccination. Among the subjects selected from the governorate of Tunis, 200 subjects were chosen at random for the additional cellular immunity study. The National Observatory of New and Emerging Diseases as well as the regional offices of different governorates ensured outgoing calls to selected vaccinated citizens eligible for the study. These structures obtained a pre-agreement of 501 subjects and filled a questionnaire that was prepared by the investigators. The study population consisted of 368 subjects who were included for the humoral antibody assessment (anti-S and anti-N antibody measurement) and 133 subjects who consented for both humoral and cellular immunity analyses.

### 2.3. Sampling

The peripheral blood samples were collected after a minimum of 3 weeks and a maximum of 7 weeks after the second dose of the vaccine. Five milliliters of whole blood were collected in a tube without anticoagulant for the serology. One milliliter of whole blood was collected in each of the 4 heparinized whole blood tubes dedicated to the cellular study (Qiagen).

### 2.4. Peripheral Anti-N and Anti-S Antibody Measurement

Sera underwent the measurement of the total anti-RBD (Receptor Binging Domain) specific antibodies by the commercial test Elecsys^®^ Anti-SARS-CoV-2 S (Cat number 09,289,267,190, Roche^®^ Diagnostic, Switzerland). The analyses were also carried out on the Cobas^®^ e411 analyzer. This test quantifies the total specific antibodies (mainly IgG with IgM and IgA) directed against the RBD protein. This test was calibrated against the 1st WHO international standard 20/136 from the National Institute for Biological Standards and Control, UK. The Elecsys^®^ Anti-SARS-CoV-2 S has obtained the FDA EUA on 25 November 2020. The sensitivity of the test is 98.8% (95%CI 98.1–99.3%) and the specificity is 100% according to the manufacturer. The obtained results are expressed by IU/mL which corresponds to 0.972 × Binding Antibody Unit per mL. Two cut-offs were proposed, namely the sensitivity cut-off, equal to 0.8 U/mL, indicating a previous contact with the virus, and the neutralizing antibodies cut-off, equal to 15 U/mL. Sera with levels higher than 15 U/mL indicate the presence of neutralizing antibodies with a positive predictive value of 100% according to the manufacturer. All sera were first analyzed without dilution. When the test indicated a result higher than the upper limit of quantification which is 250 IU/mL, the sera was diluted at 1/10 and precise level was obtained after multiplying by the dilution factor. However, when diluted sera are still quantified as higher than 250 IU/mL, the result is retained as higher than 2500 IU/mL.

In some experiments, sera were also tested for the detection of the total anti-N specific antibodies by the commercial test Elecsys^®^ anti-SARS-CoV2 qualitative assay (Cat number 09203095119, Roche^®^ Diagnostic, Switzerland). This test is a qualitative assay that measures mainly the presence of specific IgG anti-N antibodies (along with the IgM and IgA) on the Cobas^®^ e411 analyzer which is a high-throughput automated electro-chemiluminescence immunoassay. This test is the unique WHO Emergency Use Listed serology test since 7 December 2020 and it has achieved the FDA emergency use authorization on 5 February 2020. The results are expressed by index as follows: when the signal sample/cutoff is lower than 1, this indicates that sera are negative for anti-SARS-CoV-2 antibodies, however, when the signal sample/cutoff is equal to or higher than 1, this indicates that sera are positive for anti-SARS-CoV-2 antibodies.

The manufacturer’s instructions were strictly followed for both analyses

### 2.5. Cellular Immunity Analysis

The CD4 and CD8 T cell responses were evaluated using the QuantiFERON SARS-CoV-2 from Qiagen (Cat number 626115 for QuantiFERON^®^ SARSCoV-2 Starter Set Blood Collection Tubes). This assay consists of four antigen tubes, Nil, Mitogen, SARS-CoV-2 Ag1 and SARS-CoV-2 Ag2. Nil and Mitogen BCTs are intended to be used as negative and positive controls, respectively. SARS-CoV-2 Ag1 and Ag2 use a combination of antigen peptides specific to SARS-CoV-2 to stimulate lymphocytes involved in cell-mediated immunity in heparinized whole blood. The SARS-CoV-2 Ag1 tube contains CD4 + epitopes derived from the S1, S2 and RBD subunits of the Spike protein. The SARS-CoV-2 Ag2 tube contains CD4 + and CD8 + epitopes from the same antigens. Samples were processed according to manufacturer’s guidelines. IFN-γ concentration in IU/mL was then measured in the plasma from the stimulated samples by enzyme-linked immunosorbent assay (ELISA). Positive response for SARS-CoV-2 Ag1 and Ag2 (calculated as SARS-CoV-2 Ag value–Nil value) was defined by the manufacturer as a value > 0.15 IU/mL.

### 2.6. Statistical Analyses

Statistical analyses were performed using SPSS software. We described qualitative data using numbers and percentages and quantitative parameters by calculating the median (2nd quartile) and interquartile range (1st quartile–3rd quartile). The Chi2 test was used to compare percentages, the Student T test to compare 2 means for normal distributed quantitative parameters and the Mann Whitney test otherwise. To compare the immune response between different vaccines, we used the ANOVA and the Kruskal–Wallis tests according to the number of groups of comparison. Correlations between 2 quantitative variables were investigated using Pearson and Spearman correlation coefficients. The level of significance for all tests was set up at 5%.

## 3. Results

A total of 501 consented individuals were enrolled in the study. All of them were aged 40 and over and had received two doses of one of the six vaccines included in this study. There were 224 men and 277 women with a median age of 51 years (IQR 47–58 years). According to the received vaccine, they were distributed as follows: 95 received the AstraZeneca vaccine (Vaxzevria), 86 received Sputnik V from Gamaleya Research Institute, 63 received Spikevax from Moderna, 119 received Comirnaty from Pfize-BioNTech, 55 received Coronavac from Sinovac, and 83 received BIBP from Sinopharm (Table 1). For convenience, all vaccines will be referred to by their producing company, i.e., Astrazeneca, Moderna, Pfize-BioNTech, Sinovac, and Sinopharm, except for Sputnik V. The most frequent co-morbidities were diabetes and arterial hypertension (*n* = 93; 18.6% for each one), followed by obesity (*n* = 43; 8.6%), asthma (*n* = 25; 5%) and cardiovascular diseases (*n* = 23; 4.6%) (Table 1). Other comorbidities were found infrequently, such as chronic liver disease (1.6%), chronic kidney disease (1.2%), chronic lung disease other than asthma (0.8%), or chronic hematopoitic disorders (0.4%) (Data not shown).

The specific immune response against SARS-CoV-2 antigens developed after 3–7 weeks post-vaccination and consisting of anti-S antibodies along with the CD4 and CD4 and CD8 T cell responses varied significantly between all six groups (*p* < 0.0001 using Kruskal–Wallis test for all parameters, Table 2).

### 3.1. Antibody Development after SARS-CoV-2 Vaccination

**Figure 1:**
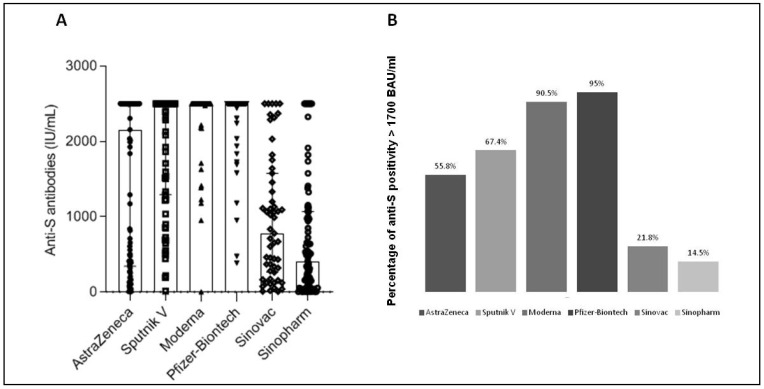
Humoral anti-S immunity in individuals vaccinated with SARS-CoV-2 vaccines. Five hundred and one people over 40 who received the vaccine from AstraZeneca (*n* = 95), Gamaleya Research Institute, Sputnik V (*n* = 86), Moderna (*n* = 63), the Pfizer-BioNTech (119), Sinovac (*n* = 55) or Sinopharm (*n* = 83) were included in this work. Anti-S antibodies were quantified in the peripheral blood at 3 to 7 weeks post-vaccination (**A**) Results are expressed in IU/mL. Along with dot plots, median with interquartile range are shown. (**B**) Percentage of positivity over the threshold of 1700 BAU/mL (corresponding to 1748 IU/mL) for anti-S antibodies is shown.

Although most vaccinated subjects developed significant levels of anti-S antibodies (>15 IU/mL) (Table 3), such levels varied greatly from one vaccine to another (Table 2). RNA vaccines gave the highest levels of anti-S antibodies (median level of 2500 IU/mL for both Moderna and Pfizer vaccines) followed by adenovirus vaccines (median level of 2157 IU/mL and 2500 IU/mL for AstraZeneca and Sputnik V, respectively) and then by inactivated vaccines (median level of 770 IU/mL and 406 IU/mL for Sinovac and Sinopharm, respectively) (Table 2 and Figure 1A). The differences were highly significant between the RNA vaccines and the other two platforms, but also between the adenovirus vaccines and the inactivated vaccines (*p* < 0.0001) (Table 4). Vaccines using the same platform induced similar levels of anti-S antibodies, except in the case of adenovirus vaccines, where we noticed significantly higher rates for the Sputnik vaccine compared to the AstraZeneca vaccine (*p* = 0.003, Table 4).

By setting the threshold for anti-S antibodies at 1700 BAU/mL (corresponding to 1748 IU/mL), a threshold shown to be correlated with total protection by Dimeglio et al. [5], we also noted significant differences between the various vaccine groups. The percentage of subjects with anti-S antibodies > 1700 BAU/mL were 95.0%, 90.5%, 67.4%, 55.8%, 21.8% and 14.5% for Moderna, Pfizer, Sputnik V, AstraZeneca, Sinovac and Sinopharm vaccines respectively (*p* < 0.0001) (Figure 1B). No difference was noted between vaccines using the same platform (*p* > 0.05), but significant differences were shown between the different platforms with positivity percentages of 93.4%, 61.3%, and 17.4% for RNA vaccines, adenovirus vaccines, and inactivated vaccines, respectively (*p* < 0.0001) (Data not shown).

### 3.2. Cellular Immune Response Development after SARS-CoV-2 Vaccination

**Figure 2:**
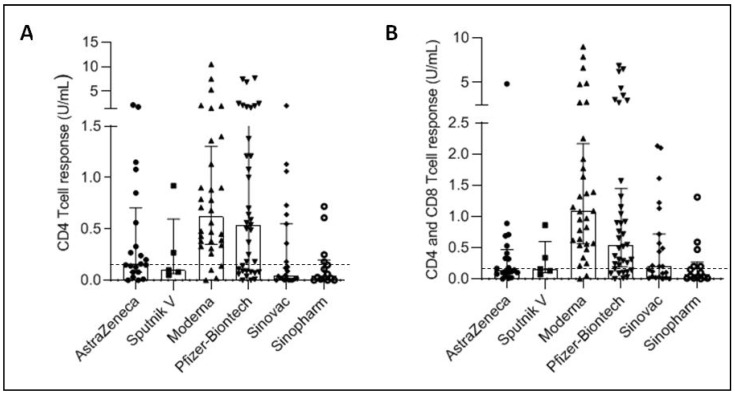
Cellular anti-S immunity in individuals vaccinated with SARS-CoV-2 vaccines CD4 (**A**) and CD4 and CD8 (**B**) T cell responses were quantified in the sera of individuals who received the vaccine from AstraZeneca (*n* = 95), Gamaleya Research Institute, Sputnik V (*n* = 86), Moderna (*n* = 63), Pfizer-BioNTech (119), Sinovac (*n* = 55) and Sinopharm (*n* = 83). Results are expressed in IU/mL. Along with dot plots, median with interquartile range are shown. The cut-off of positivity (0.15 IU/mL) is indicated.

The induction of a cellular response to protein S appears to considerably vary from one vaccine to another either quantitatively (Table 2 and Figure 2) or qualitatively (Table 3). Again, the highest levels and frequencies were found with RNA vaccines followed by adenovirus vaccines and then inactivated vaccines. The differences were not significant between vaccines using the same platform (Table 4). Yet, the level of the cellular response was significantly greater in people who received RNA vaccines compared to those who received the other two platforms. Unexpectedly, there was no significant difference between the level of response induced by adenovirus vaccines and that induced by inactivated vaccines (Table 4).

### 3.3. Correlation between Humoral and Cellular Responses Post-SARS-CoV-2 Vaccination

**Figure 3:**
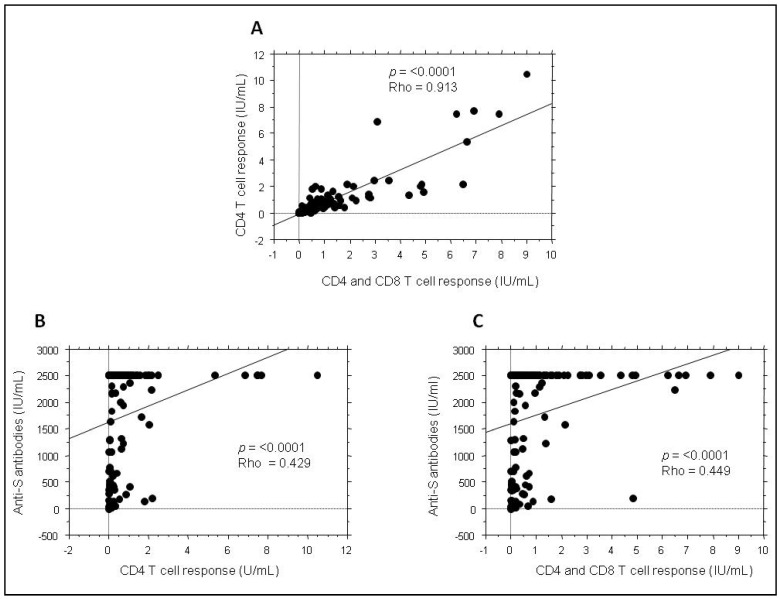
Correlation between humoral and cellular anti-S immunity in subjects vaccinated with SARS-CoV-2 vaccines. Anti-S antibodies and CD4 and CD4/CD8 T cell responses were quantified within the total cohort. Correlations between the three types of immune responses were analyzed with Spearman test: (**A**) between anti-CD4 and anti-CD4 and CD8 T cell response, (**B**) between anti-S antibodies and CD4 T cell response and (**C**) between anti-S antibodies and CD4 and CD8 T cell response.

While the CD4 and CD8 cellular responses were, as expected, strongly correlated (*p* < 0.0001 with a Rho = 0.913) (Figure 3A), the humoral anti-S response was significantly correlated with the cellular anti-S response but in a way which does not seem to be linear probably due the fact that the anti-S antibodies are capped at 2500 IU/mL (Figure 3B,C). Even when values over 2500 IU/mL were removed, the correlation analysis did not show better linearity (Rho = 0.398 and Rho = 0.401 for anti-CD4 and anti-CD4 and CD8 response, respectively) (Data not shown). Finally, by analyzing the correlation between humoral and cellular responses for each type of vaccine, we noticed that it was only significant for inactivated vaccines (Table 5).

### 3.4. Association of the Post-Vaccination Immune Response with Different Clinical Parameters

For the entire study population, no correlation was found between age and the post-vaccination immune responses (data not shown). No association was found with the gender or the presence of comorbidities (Table 6).

### 3.5. Anti-N Antibodies in Subjects Who Received Inactivated Vaccines

**Figure 4:**
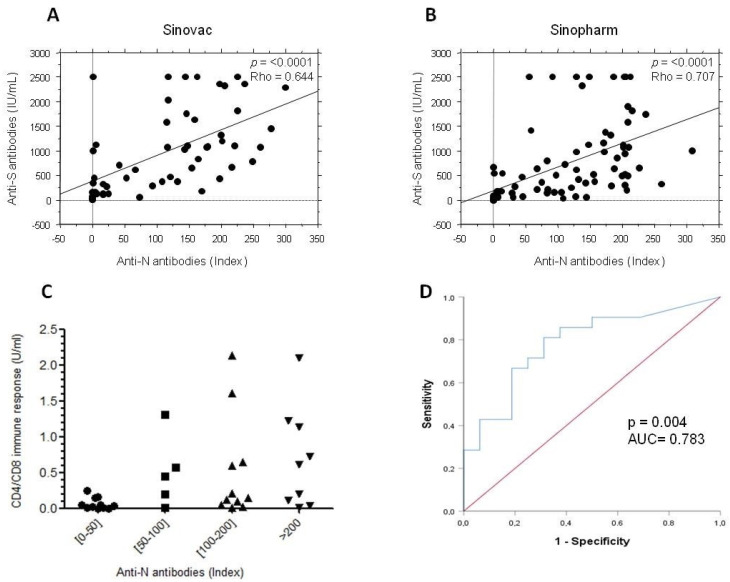
Anti-N immunity in individuals who received inactivated SARS-CoV-2 vaccines. Anti-N antibodies were quantified in the sera of individuals vaccinated with Sinovac (*n* = 55) and Sinopharm (*n* = 83). Correlation between anti-N and anti-S antibodies in subjects vaccinated with Sinovac (**A**) or Sinopharm (**B**) are shown. (**C**) Positive CD4/CD8 immune response consisting of positivity of CD4 and/or CD4 and CD8 T cell responses is shown according to the range of anti-N antibody index. (**D**) The ROC curve predicting the positivity of CD4/CD8 immune response according to the positivity or negativity of anti-N antibody is shown. The area under curves (AUC) and the *p* value of the ROC curve are indicated.

Since SARS-CoV-2 inactivated vaccines also induce antibodies against nucleocapsid protein (N antibodies), these antibodies were checked in subjects who received Sinovac or Sinopharm. Nearly 80% of subjects who received inactivated vaccines developed anti-N antibodies (data not shown). For both vaccines, the level of anti-N antibodies was significantly correlated with the level of anti-S antibodies (Figure 4A,B). Interestingly, the positivity of the cellular anti-S response was more frequent in subjects who exhibited high indexes of anti-N antibodies. Consistently, all people who did not develop anti-N antibodies did not show any cellular response (Figure 4C). In order to verify from which titer of anti-N antibody the cellular response becomes positive, the vaccinees were stratified in two groups according to the positivity or negativity of the cellular immune response. A ROC curve was then designed by analyzing the anti-N antibody levels in these two groups. The ROC curve (area under the curve of 0.783 with a *p* = 0.004) showed that anti-N antibody index over 48.7 was significantly associated with a positive development of a cellular anti-S response (86% of sensitivity and 80% of specificity) and could thus be predictive of a previous SARS-CoV-2 infection (Figure 4D).

### 3.6. Antibody and Cellular Immunity in People with Negative Anti-N Antibodies

**Figure 5:**
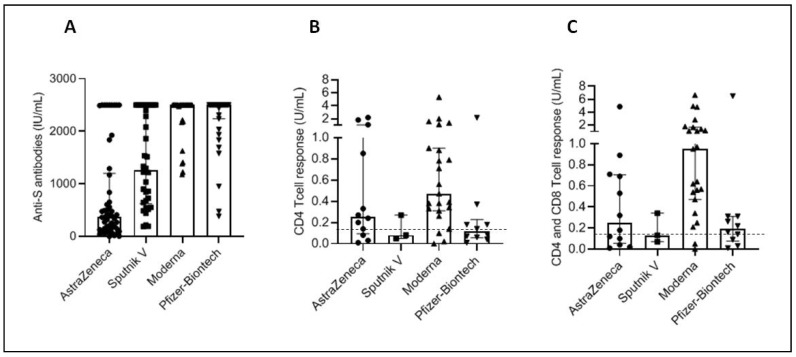
Humoral and cellular anti-S immunity in vaccinees with negative anti-N antibodies. Anti-S antibodies (**A**) and CD4 (**B**) and CD4 and CD8 (**C**) T cell responses were quantified in the peripheral blood at 3 to 7 weeks post-vaccination in subjects who were negative for anti-N antibodies (*n* = 50 for AstraZeneca, *n* = 34 for Sputnik V, *n* = 37 for Moderna and *n* = 38 for Pfizer-BioNTech). Results are expressed in IU/mL. Along with dot plots, median with interquartile range are shown. The cut-off of positivity of the cellular immunity (0.15 IU/mL) is indicated in panel (**B**,**C**).

Since an asymptomatic infection with SARS-CoV-2 could also not be excluded in vaccinees who received RNA- or adenovirus-based vaccines, the humoral and cellular immunity has been then reanalyzed in people with negative anti-N antibodies (*n* = 50 for AstraZeneca, *n* = 34 for Sputnik V, *n* = 37 for Moderna and *n* = 38 for Pfizer-BioNTech). Similar results to those of the total cohort were obtained except for the Moderna vaccine, which gave a better cellular immune response than the Pfizer vaccine and RNA vaccines, which induced similar cellular immune responses to adenovirus vaccines (Table 7 and Figure 5). Table 8 summarizes median levels of the humoral and cellular responses in all groups of vaccines according to the positivity or the negativity of the anti-N antibodies.

## 4. Discussion

This is the first study comparing the levels of both humoral and cellular immune responses induced by six different SARS-CoV-2 vaccines. Although several works have compared the immunogenicity of different vaccines, such comparisons have so far been limited to 2–4 vaccines and rarely concerned the cellular immune response [6,7,8,9,10,11,12,13,14,15,16,17,18,19]. The study was limited to subjects over 40 years of age for a better comparability. Indeed, indication of the AstraZeneca vaccine in Tunisia being restricted to subjects over 40 years, we have chosen a comparable age group for the other vaccines. Consistently, our results clearly show, within this age group, the absence of a correlation between the immune response induced by vaccines and age.

Our results also provide a demonstration of the superiority of RNA vaccines for the induction of a specific immune response over Adenovirus-based vaccines, i.e., inactivated vaccines providing less favorable results. Such data correlate well with the efficiencies found in clinical trials and real-life studies [3]. Interestingly, vaccines of the same platform gave very similar results; the only exception being the AstraZeneca and Sputnik V vaccines. The latter giving significantly higher results in humoral response and the AstraZeneca vaccine giving slightly higher cellular responses, though differences were not significant. This could be explained by the different types of Adenoviruses used by the two vaccines (a simian Adenovirus for the first and a human type for the second). Yet unidentified factors could also be at play. Whatever the case, these results correlate with the efficacy rates of protection against infection reported during clinical trials and/or real-life studies in the two vaccines, as Sputnik V showed an efficacy of 92% against 70% for the AstraZeneca vaccine, against the SARS-CoV-2 virus [20,21].

Regarding the anti-S antibodies for SARS-CoV-2 and thanks to the use of calibrated quantitative tests, the results obtained in the different studies have become more comparable. Yet, few studies have defined a protective level of such antibodies. Dimeglio et al. showed that a partial protection was achieved when the level of induced anti-S antibodies exceeded 141 BAU/mL. Over 1700 BAU/mL, protection was considered total since no subject with this level has developed disease [5]. By setting up this protective threshold, we showed that the positivity rates differed from one vaccine to another. Indeed, the positivity rate was higher for the Moderna vaccine followed by the Pfizer vaccine, then the two Adenovirus vaccines, and finally the two inactivated vaccines. Despite these disparities, the difference between vaccines seems less pronounced than that obtained when analyzing the median antibody levels. Yet, the thresholds of 141 BAU/mL and 1700 BAU/mL should however be taken with caution as the work of Dimeglio et al. was carried out before the appearance of variants escaping the antibody response. Consistently, a more recent work by the same team demonstrated higher thresholds for Delta and Omicron infections (2905 and 6967 BAU/mL, respectively) [22].

Cellular immune response, and in particular that involving the IFN-γ- producing CD4 and CD8 T lymphocytes, is crucial for fighting against viral infections including SARS-CoV-2 infections. The cellular immune response is also associated with protection against severe forms of COVID-19 [23,24,25]. Such a response is all the more important since recent data showed it is more durable than the antibody one [26,27] and more interestingly less sensitive to genetic variations of viruses [28,29]. QuantiFERON^®^SARS-CoV-2 is a good Interferon Gamma Release Assay (IGRA) that has a proven efficacy in demonstrating a cellular CD4 and CD8 T cell response directed against the S protein of SARS-CoV-2 [30,31,32,33]. As expected, our results showed that both CD4 and CD8 T cell responses are strongly correlated. Once again, the cellular immune response was higher in the group of RNA vaccines. There was almost no difference between the level of cellular immune response induced by both Adenovirus vaccines and that induced by both of inactivated vaccines although the humoral response was significantly different between these two platforms.

Surprisingly, the humoral response was not strongly correlated with the cellular response, although both were directed against protein S. This could be due to the fact that the anti-S antibody data were capped at 2500 IU/mL However, humoral and cellular responses were well correlated in subjects who received the inactivated vaccines. One explanation could be that the levels of anti-S antibodies are at the maximum in most individuals who received RNA-and adenovirus-based vaccines which was not the case in people who received the inactivated vaccines. This could also be due to the fact that the cellular response is triggered in individuals who received inactivated vaccines mainly after breakthrough SARS-CoV-2 infection. Can we thus hypothesize that SARS-CoV-2 infection gives correlated cellular and humoral responses while the vaccine does not? Further studies are needed to confirm this. The case of Sputnik, which is the only non-inactivated vaccine to exhibit an excellent correlation between the humoral and the cellular anti-S response, also remains intriguing.

Although several co-morbidities, such as diabetes and hypertension, were noticed in subjects included in the study, at a rate of nearly 18% each, no association with the level of the humoral and cellular response directed against S protein was found.

Regarding the antibody response against the nucleocapsid protein, more than 80% of the subjects who received inactivated vaccines developed, as expected, anti-N antibodies. In this group, those who did not develop anti-N antibodies did not have any specific cellular immune response as detected by IGRA, confirming what we already know about conventional platforms, i.e., they do not induce high specific cellular responses [34]. While anti-N antibody detection may ascertain the occurrence of an old infection with SARS-CoV-2 in subjects who received RNA or adenovirus platforms, it was more difficult for those who received inactivated vaccines. Interestingly, our data showed that people vaccinated with inactivated vaccines and exhibiting anti-N antibody indexes of more than 48 had a positive cellular response which corresponded probably to subjects previously infected by SARS-CoV-2.

Although all subjects with symptomatic COVID-19 were excluded from the study, a high proportion of vaccinees who received RNA or adenovirus platforms were shown to have old asymptomatic infections with SARS-CoV-2, confirmed by the presence of anti-N antibodies. These data are consistent with the results of the national seroprevalence survey of SARS-CoV-2 infection conducted in Tunisia in March–April 2021, following the second wave just before the introduction and subsequent wide circulation of the Alpha variant in the country. The survey showed a seroprevalence of about 30% of cases with more than 90% of cases going unnoticed (manuscript in preparation). A higher proportion of asymptomatic infections is thus expected in our study, which was conducted after the fourth epidemiological wave (caused by a broad circulation of the Delta variant) of SARS-CoV-2 infections in September–October 2021. Unfortunately, precise figures in Tunisia during this period are not available. Interestingly, if we exclude individuals with positive anti-N antibodies, our data further confirm the superiority of the RNA vaccine in inducing the antibody anti-S response. Regarding the cellular immune response, the Moderna vaccine gave the best results, yet the RNA vaccines and the adenovirus vaccine gave similar results.

Obviously, our study has several limitations. The first one is the lack of data concerning neutralizing antibodies, which are known to be well correlated with protection [35,36]. Yet, several studies showed that anti-S binding antibodies are very well correlated with neutralizing antibodies [35], and therefore the adjournment of their search can be justified. The second limitation is the relatively different sizes in each category of vaccines. This is due to the random inclusion of subjects, although it did not prevent appropriate statistical analyses. The third limitation is that the anti-S antibody data were capped at 2500 IU/mL because of the relative lack of linearity of the used technique above this level. The use of a double antigen-sandwich method (Roche Elecsys immunoassays) could also be a limitation since a recent study showed that the avidity of the SARS-CoV-2 antibodies could affect the results with this type of assay [37]. Another limitation is the small number of subjects in which cellular immune response was studied. This was due to logistical issues. Despite the two latter limitations, appropriate statistical analyses were again possible and very significant differences were shown. A lack of monitoring of immune responses over time is also a limiting factor. This is a very important study to perform since the durability of the immune response could be more relevant than a cross-sectional survey. A new study cohort has already started to test this point.

Collectively, our data demonstrate the superiority of the RNA-based COVID-19 vaccines, in particular the Moderna vaccine, in terms of humoral and cellular immunogenicity. Our results comparing between different vaccine platforms in a similar population are of great importance since they may help decision makers adopt the best strategy for further national vaccination programs.

## Figures and Tables

**Table 1:** Main characteristics of the study population.

	N	Median Age (IQR)	Sex M/F	Diabetes N (%)	Hypertension N (%)	Obesity N (%)	Cardiovascular Disease N (%)	Asthma N (%)
**Astrazeneca**	95	50 (44–57)	43/52	10 (10.5)	15 (15.8)	6 (6.3)	3 (3.2)	6 (6.3)
**Sputnik V**	86	55 (50–59)	35/51	22 (26.5)	24 (27.9)	4 (4.7)	5 (5.8)	4 (4.7)
**Moderna**	63	43 (41–48)	26/37	6 (9.5)	4 (6.3)	4 (6.3)	0 (0.0)	1 (1.6)
**Pfizer-BioNTech**	119	56 (51–63)	55/64	31 (26.1)	28 (23.5)	15 (12.6)	9 (7.6)	4 (3.4)
**Sinovac**	55	55 (49–60)	26/29	10 (18.2)	10 (18.2)	5 (9.1)	2 (3.6)	4 (7.3)
**Sinopharm**	83	49 (48–51)	39/44	14 (16.9)	12 (14.5)	9 (10.8)	4 (4.8)	6 (7.2)
**All vaccines**	501	51 (47–58)	224/277	93 (18.6)	93 (18.6)	43 (8.6)	23 (4.6)	25 (5)

**Table 2:** Median levels of the humoral and cellular immune responses in the different groups of vaccines.

	N	Anti-S Antibodies Median Level [IQR]	CD4 Response Median Level [IQR]	CD4 + CD8 Response Median Level [IQR]
**Astrazeneca**	95	2157.00 [340.9–2500.00]	0.16 [0.08–0.70]	0.14 [0.09–0.47]
**Sputnik V**	86	2500.00 [1294.50–2500.00]	0.10 [0.065–0.595]	0.16 [0.10–0.60]
**Moderna**	63	2500.00 [2500.00–2500.00]	0.62 [0.35–1.30]	1.08 [0.56–2.16]
**Pfizer**	119	2500.00 [2500.00–2500.00]	0.54 [0.09–1.51]	0.55 [0.19–1.44]
**Sinovac**	55	777.60 [261.00–1575.00]	0.05 [0.01–0.55]	0.20 [0.03–0.72]
**Sinopharm**	83	409.50 [60.88–1066.00]	0.045 [0.0075–0.1975]	0.075 [0.010–0.267]
***p* value**		**<0.0001**	**<0.0001**	**<0.0001**

**Table 3:** Percentage of positivity of the immune responses obtained in the different groups of vaccine.

	Anti-S Antibodies % of Positivity (>15 IU/mL)	CD4 Response % of Positivity (>0.15 IU/mL)	CD4 + CD8 Response % of Positivity (>0.15 IU/mL)
**Astrazeneca**	97.9	47.6	38.1
**Sputnik V**	98.8	40.0	40.0
**Moderna**	100	87.5	93.8
**Pfizer**	100	62.2	75.7
**Sinovac**	96.4	30.4	52.2
**Sinopharm**	85.5	21.4	35.7

**Table 4:** Comparison of the immune responses between different vaccines (*p* value).

	Anti-S Antibodies	CD4 Response	CD4 + CD8 Response
**Astrazeneca** ***vs.*** **Sputnik V**	0.003	0.431	0.056
**Moderna** ***vs.*** **Pfizer**	0.497	0.828	0.275
**Sinovac** ***vs.*** **Sinopharm**	0.054	0.828	0.275
**RNA vaccines** ***vs.*** **Adenovirus vaccines**	**<0.0001**	**0.002**	**<0.0001**
**RNA vaccines** ***vs.*** **Inactivated vaccines**	**<0.0001**	**<0.0001**	**<0.0001**
**Adenovirus vaccines** ***vs.*** **Inactivated vaccines**	**<0.0001**	0.206	0.838

**Table 5:** Correlation between humoral and cellular anti-S immune response.

	Anti-S *vs.* CD4 Response	Anti-S *vs.* CD4 + CD8 Response
**Astrazeneca**	*p* = 0.135	*p* = 0.161
**Sputnik V**	*p* = 0.554	*p* = 0.605
**Moderna**	*p* = 0.605	*p* = 0.605
**Pfizer**	*p* = 0.521	*p* = 0.536
**Sinovac**	***p* = 0.006** ***r* = 0.552**	***p* = 0.017** ***r* = 0.449**
**Sinopharm**	***p* = 0.015** ***r* = 0.631**	***p* = 0.013** ***r* = 0.642**

**Table 6:** Mean values of the immune response according to the sex and comobidities.

	Sex (M/F)	Diabetes (Yes/No)	Hypertension (Yes/No)	Obesity (Yes/No)	Cardiovascular Disease (Yes/No)	Asthma (Yes/No)
**Anti-S Antibodies** **UI/mL**	1624/1771 (*p* = 0.096)	1712/1704 (*p* = 0.943)	1709/1705 (*p* = 0.969)	1872/1690 (*p* = 0.219)	1644/1709 (*p* = 0.776)	1965/1692 (*p* = 0.136)
**CD4 T Cell Response** **UI/mL**	0.753/0.903 (*p* = 0.598)	0.764/0.849 (*p* = 0.822)	1.619/0.734 (*p* = 0.179)	0.500/0.840 (*p* = 0.618)	1.943/1.782 (*p* = 0.367)	0.858/0.833 (*p* = 0.947)
**CD4 + CD8 T Cell Response** **UI/mL**	0.948/1.131 (*p* = 0.540)	0.746/1.108 (*p* = 0.275)	1.372/1.006 (*p* = 0.451)	0.450/1.057 (*p* = 0.390)	1.955/1.004 (*p* = 0.366)	1.311/1.033 (*p* = 0.689)

**Table 7:** Comparison of the immune responses between mRNA and adenovirus-based vaccines in individuals with negative N antibodies (*p* value).

	Anti-S Antibodies	CD4 Response	CD4/CD8 Response
**Astrazeneca** ***vs.* Sputnik V**	**0.0001**	0.311	0.663
**Moderna** ***vs.* Pfizer**	0.595	**0.011**	**0.006**
**RNA vaccines** ***vs.* Adenovirus vaccines**	**<0.0001**	0.379	**0.049**

**Table 8:** Median levels of the humoral and cellular immune responses in the different groups of vaccines according the positivity or the negativity of the anti-N antibodies.

		Positive Anti-N Antibodies			Negative Anti-N Antibodies	
	Anti-S Antibodies Median Level [IQR]	CD4 Response Median Level [IQR]	CD4 and CD8 Response Median Level [IQR]	Anti-S Antibodies Median Level [IQR]	CD4 Response Median Level [IQR]	CD4 and CD8 Response Median Level [IQR]
**Astrazeneca**	2500.00 [2500.00–2500.00]	0.15 [0.04–0.36]	0.12 [0.09–0.25]	376.45 [144.975–1201.50]	0.255 [0.095–1.022]	0.25 [0.055–0.705]
**Sputnik V**	2500.00 [2500.00–2500.00]	0.51 [0.10–0.62]	0.51 [0.16–0.61]	1261.50 [622.15–2500.00]	0.08 [0.05–0.29]	0.13 [0.07–0.34]
**Moderna**	2500.00 [2500.00–2500.00]	0.90 [0.456–4.81]	1.92 [1.07–5.33]	2500.00 [2488.00–2500.00]	0.47 [0.31–0.90]	0.95 [0.47–1.64]
**Pfizer**	2500.00 [2500.00–2500.00]	0.70 [0.25–1.82]	0.89 [0.37–2.74]	2500.00 [2239.00–2500.00]	0.115 [0.055–0.227]	0.19 [0.075–0.31]
**Sinovac**	1026.00 [367.95–1696.50]	0.09 [0.02–0.64]	0.21 [0.04–1.13]	35.89 [8.61–94.025]	0.025 [0.0025–0.10]	0.05 [0.00–0.137]
**Sinopharm**	530.70 [209.10–1153.00]	0.06 [0.02–0.25]	0.10 [0.03–0.47]	5.10 [2.69–15.92]	0.000 [0.0000–0.000]	0.01 [0.000–0.01]
***p* value**	**<0.0001**	**0.001**	**<0.0001**	**<0.0001**	**0.003**	**0.001**

## Data Availability

Not applicable.
